# Pseudohypoaldosteronism type 1b in fraternal twins of a Chinese family: report of two cases and literature review

**DOI:** 10.20945/2359-3997000000620

**Published:** 2023-05-10

**Authors:** Zhen Gao, Jingjing Sun, Cheng Cai, Xiaohui Gong, Li Ma

**Affiliations:** 1 Shanghai Jiao Tong University School of Medicine Shanghai Children's Hospital Shanghai China Department of Neonatology, Shanghai Children's Hospital, School of Medicine, Shanghai Jiao Tong University, Shanghai, China; 2 Shanghai Key Laboratory of Embryo and Reproduction Engineering NHC Key Laboratory of Medical Embryogenesis and Developmental Molecular Biology Shanghai China NHC Key Laboratory of Medical Embryogenesis and Developmental Molecular Biology & Shanghai Key Laboratory of Embryo and Reproduction Engineering, Shanghai, China

## Abstract

Here, we report the clinical observations of two Chinese fraternal twins who presented with severe dehydration, poor feeding, and absence of stimuli responses within a few days of birth. Trio clinical exome sequencing of the family identified compound heterozygous intronic variants (c.1439+1G>C and c.875+1G>A) in *SCNN1A* gene in these two patients. Sanger sequencing results showed that the c.1439+1G>C variant was inherited from the mother, and c.875+1G>A from the father, rarely reported in pseudohypoaldosteronism type 1 with sodium epithelial channel destruction (PHA1b) patients. Case 2 received timely symptomatic treatment and management after obtaining these results, which improved the clinical crisis. Our results suggest that the compound heterozygous splicing variants in *SCNN1A* were responsible for PHA1b in these Chinese fraternal twins. This finding extends the knowledge of the variant spectrum in PHA1b patients and highlights the application of exome sequencing in critically ill newborns. Finally, we discuss supportive case management, particularly in maintaining blood potassium concentration.

## INTRODUCTION

Pseudohypoaldosteronism type 1 (PHA1) is a rare heterogeneous syndrome characterized by transepithelial sodium transport deficiency ( 1 ). Typical clinical manifestations of PHA1 include failure to thrive, hyperkalemia, hyponatremia, metabolic acidosis accompanied by elevated plasma aldosterone, and renin levels in the neonatal period ( 2 ). PHA1 is classified as either renal PHA, an autosomal dominant type (MIM # 177735), or systemic PHA1, an autosomal recessive form (MIM # 264350), also known as multiple target organ defects. Renal pseudohypoaldosteronism type 1 with renal tubular resistance (PHA1a) is caused by variants in *NR3C2* gene, which encodes the mineralocorticoid receptor, that decrease sodium reabsorption and potassium secretion in renal tubules. The clinical symptoms of salt loss in PHA1a can gradually improve with age ( 3 ). The systemic PHA1(PHA1b) is caused by mutations in subunits of the epithelial sodium channel (ENaC): alpha subunit (SCNN1A), beta subunit (SCNN1B), or gamma subunit (SCNN1G) ( 4 ). Patients with PHA1b present a more severe clinical phenotype than those with PHA1a, including salt-losing crises and life-threatening hyperkalemia. PHA1b typically presents in the neonatal period with severe dehydration, vomiting, and failure to thrive. It does not spontaneously improve during early childhood ( 5 – 6 ).

The epithelial sodium channel (ENaC) allows sodium transportation from the lumen into the epithelial cell and modulates sodium in the extracellular fluid ( 7 ). Transportation of sodium via ENaC is the rate-limiting step in sodium reabsorption in the apical membrane of epithelia ( 8 ). ENaC is comprised of subunits: alpha subunit (encoded by *SCNN1A* ), beta subunit (encoded by *SCNN1B* ), or gamma subunit (encoded by *SCNN1G* ). Variants in these genes lead to significant salt loss from sweat, saliva, stool, and urine ( 9 ) and cause systemic PHA1b ( 4 ). However, the primary phenotype of PHA1b is easily confused with other endocrine disorders such as congenital adrenal hyperplasia (CAH) associated with 21-hydroxylase deficiency or 3-beta-hydroxysteroid dehydrogenase deficiency, hypoaldosteronism due to aldosterone deficiency, and antenatal or infantile Bartter syndrome. Such confusion delays a diagnosis when prompt treatment of PHA1b is essential, as it may be fatal due to salt depletion and high blood potassium levels ( 10 ).

This study reports on two fraternal Chinese twin patients with presumed neonatal clinical PHA1b. Both children carried heterozygous splicing variants c.875+1G>A and c.1439+1G>C in the *SCNN1A* gene. The variants were identified with trio clinical exome sequencing (Trio-CES sequencing) and verified by sanger sequencing. Both children showed similar severe clinical phenotypes, including feeding difficulties, dehydration, and failure to thrive. The second twin diagnosed had significant improvement by conventional treatment after getting the genetic test result. Additionally, we review and summarize *SCNN1A* variants implicated in PHA1b, which provides a convenient resource for clinicians. Finally, we offer clinical symptoms and treatment of PHA1b during the neonatal period.

## CASE REPORT

### Case 1 (Female Twin Proband)

An eight-day-old female newborn was admitted to the neonatal intensive care unit (NICU) due to poor feeding and absence of responses to stimuli. The patient was delivered by cesarean section at full term gestation with a birth weight of 2900 g (>P10) without any prenatal problems. She was the sixth child of non-consanguineous parents with three sisters, an older brother, and her twin brother. Her older brother died shortly after he was born. Her parents were healthy with no relevant family history.

On admission, her physical examination revealed confusion, poor response to stimuli, no crying, pale complexion, weak heart sounds, and weakened apical beats. Her weight had decreased to 2620 g (9.7% of weight loss) without changes in her skin pigmentation. She had normal genitalia without clitoromegaly, labial fusion or hyperpigmentation. Physical examination showed a weak heart rate of 50 beats per minute and her blood pressure couldn't be measured. Her laboratory results were: blood glucose (9.3 mmol/L), hyponatremia (117 mmol/L), hyperkalemia (10.2 mmol/L), chloride ion (90 mmol/L), metabolic acidosis (pH 7.0), HCO3- (14.2 mmol/L), PCO2 (6.1 kPa), and elevated lactic acid (11.4 mmol/L). Her echocardiography showed an atrial septal defect of 0.2 cm and a patent ductus arteriosus of 0.1 cm. Her X-ray revealed pneumonia, and her renal and abdominal ultrasounds were normal. The patient's metabolic newborn screening was negative. Additional laboratory results were: cortisol (671.86 nmol/L), 17-hydroxyprogesterone (7.8 ng/mL), testosterone (3.87 nmol/L), adrenocorticotropic hormone (ACTH) (3.75 pmol/L), and evaluated aldosterone (>2,000 ng/L). These findings rule out the possibility of CAH ( Table 1 ).

**Table 1:** Clinical, biochemical, and endocrine profiles of Chinese twins with a systemic form of PHA1

Patients	Case 1	Case 2	Reference range
Sex	Female	Male	
Birth	Term	Term	
Birth weight (g)	2.800	3.100	
Consanguinity of family history	No	No	
Day of onset	8	9	
Primary symptoms	Poor response, refusing milk Heterozygous intronic missense mutations in SCNN1A c.1439+1G>C and c.875+1G>A	Poor response, refusing milk Heterozygous intronic missense mutations in SCNN1A c.1439+1G>C and c.875+1G>A	
Serum HCO3 (mmol/L)	12.9	11.3	18-23
Serum Creatine (μmol/L)	67	87	27-66
Serum Sodium (mmol/L)	117	127	136-145
Serum Potassium (mmol/L)	10.4	10.1	3.5-5.2
PH	7.09	7.06	7.33-7.49
BE (mmol/L)	-15.8	-26.7	-3-3
Lactose (mmol/L)	11.4	9.7	0.5-2.2
Aldosterone (ng/L)	>2000	1337.18	59.5-173.9
Plasma renin activity (μg/dL/h)	N/A	9.47	0-15
ACTH (pmol/L) 8am	3.75	8.5	1.59-14
Cortisol (nmol/L)	671.86	348.03	185.00-624.00
Renal ultrasound	Normal	Normal	
Testosterone (nmol/L)	3.87	4.82	
17-OHP(ng/mL)	7.8	9.27	0-11
Management		When discharged from hospital, he was intake with 10% NaCl at 10 mEq/kg/d and ionexchange resins at 1 g/kg/d	
Complication	Died on day 2 of admitted in hospital due to cardiac arrest	Died at 5 months after birth due to cardiac arrest	

After admission, the patient was given cardiopulmonary resuscitation, emergency tracheal intubation, two normal saline boluses, and intravenous adrenaline. Then, the girl received NaHCO3, 10% NaCl, insulin drip, and a calcium gluconate injection to correct metabolic acids and electrolyte abnormalities. This treatment was ineffective. After 12 hours, the patient developed ventricular tachycardia requiring cardioversion due to hyperkalemia (K+ = 9.8 mmol/L). The patient's parents were informed of the critical condition, and we suggested continuous renal replacement therapy (CRRT), which her parents refused. The baby's condition deteriorated, and she passed away 26 hours after admission due to cardiac arrest.

### Case 2 (Male Twin Proband)

The second patient was the twin brother of case 1. One day after his younger sister died, the boy was sent to the emergency room due to similar symptoms. Upon arrival, the baby received cardiopulmonary resuscitation. After the resuscitation, his heart rate rose to 90 beats per minute, and he was sent to the NICU.

Similar to his sister, the boy had no specific facial features and normal male genitalia. The boy's birth weight was 3100 g (P10), which had decreased to 2400 g (22.6% of weight loss) when admitted to the NICU. As with his sister, he had no known prenatal abnormality. Physical examination showed heart rate of 120 beats per minute and his blood pressure was 70/40 mmHg. After his admission, we ordered a series of laboratory and imaging examinations. We found that the twins’ laboratory examinations were similar. His laboratory examination showed hyponatremia (125 mmol/L), hyperkalemia (7.3 mmol/L), chloride ion (104 mmol/L), metabolic acidosis (pH 6.86), HCO3^–^ (6.8 mmol/L), PCO_2_ (5.1 kPa), elevated lactic acid (11.0 mmol/L), and blood glucose (6.5 mmol/L). His echocardiography showed an atrial septal defect of 0.3 cm and decreased left ventricular systolic and diastolic function. The X-ray revealed pneumonia, and his renal and abdomen ultrasounds were normal. The patient's metabolic newborn screenings were negative.

**Figure 1:**
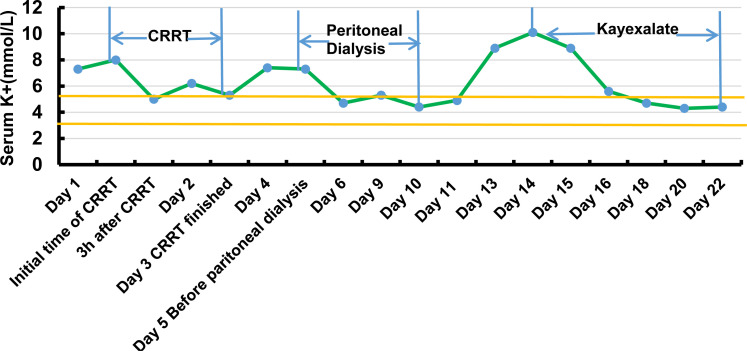
Serum potassium levels of case 2 during the first hospitalization.

After being admitted to NICU, he was given mechanical ventilation, two normal saline boluses, and intravenous adrenaline. At the same time, the boy was given NaHCO3, 10% NaCl, insulin drip, and calcium gluconate injection to correct metabolic acids and electrolyte abnormalities. However, the treatment was not effective. After four hours, the baby developed ventricular tachycardia and a complete left bundle branch block requiring cardioversion due to hyperkalemia (12.0 mmol/L). With the parents’ consent, we administered CRRT for two days. Soon, his electrolytes were within normal limits. However, when CRRT was discontinued, the boy's serum potassium level rose. We then started peritoneal dialysis to maintain his potassium level. His potassium was within normal limits during peritoneal dialysis, and the boy was given oral feeding with NaCl ( Figure 1 ).

**Figure S1:**
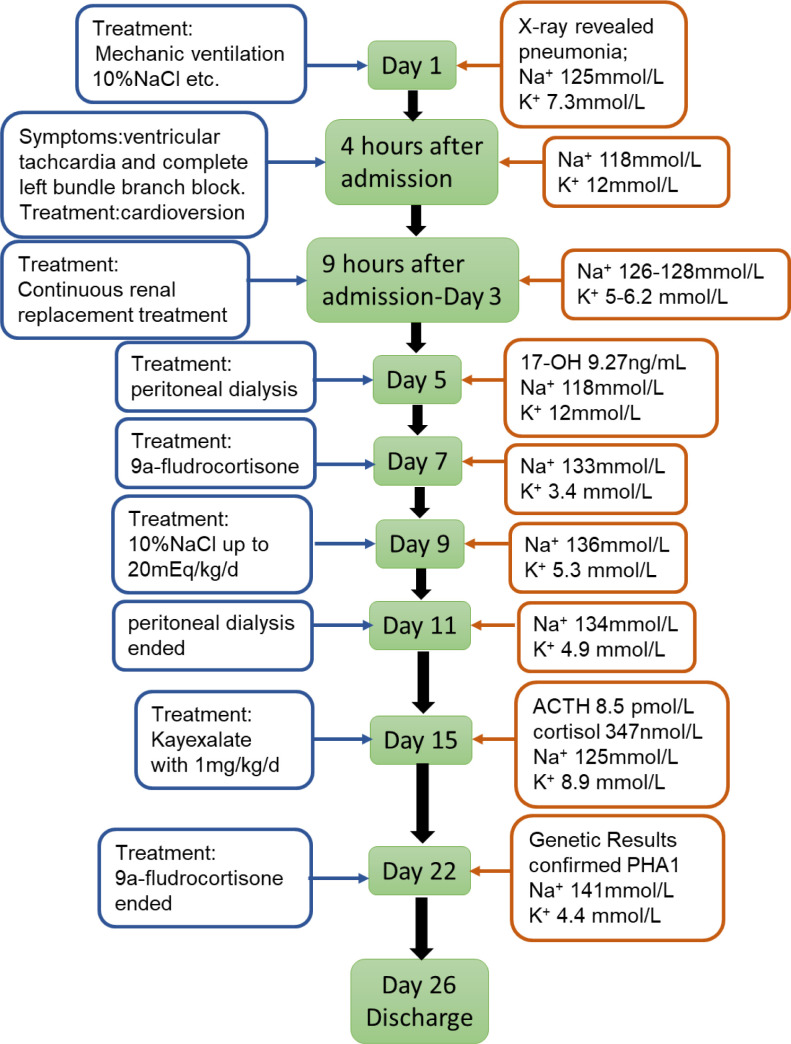
Timeline of case 2.

In light of his clinical manifestations, we initially suspected CAH. However, 9a-fludrocortisone supplementation therapy for two weeks was ineffective ( Figure S1 ).

We performed additional laboratory tests for cortisol level (348.03 nmol/L), 17-hydroxyprogesterone (9.27 ng/mL), testosterone (4.82 nmol/L), ACTH (8.5 pmol/L), plasma renin activity (9.47 μg/dL/h), and aldosterone (1337.18 ng/L) which excluded the possibility of CAH ( Table 1 ).

**Figure 2:**
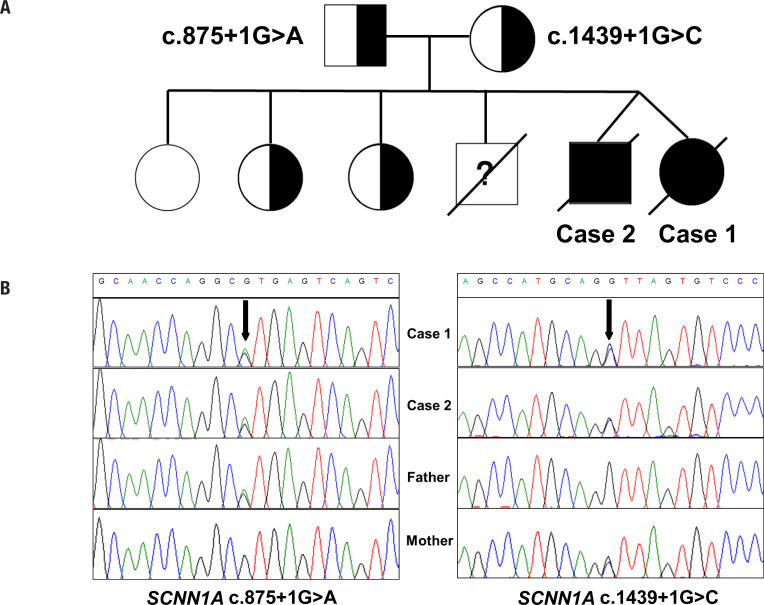
Genetic findings. ( **A** ) Pedigree of the family with segregation of the identified *SCNN1A* variants. The squares represent males, and circles represent females. The filled symbols indicate the affected individuals. ( **B** ) Sanger sequencing verification results. Black arrows indicate the point variants.

As PHA1b was suspected, trio clinical exome sequencing was performed to identify the potential pathogenic variants and exon Del/Dup analysis in related genes. Therefore, genomic DNA was extracted from peripheral blood lymphocytes from the patient and her parents. Exome capture was carried out using the Trusight Rapid Capture (Illumina), and targeted sequencing was performed on a Hiseq 4500 platform (Illumina) by using pair-end reads. Data processing, sequence alignment to GRCh37, and variant filtering and ranking by allele frequency, were conducted to predict functional impact in accordance with the classifications of Human Gene Mutation Database (HGMD). Using Trio-CES sequencing, we found that both patients had compound heterozygous variants in the *SCNN1A* gene (c.1439+1G>C and c.875+1G>A), which are responsible for PHA1b. The former was inherited from the mother and the latter from the father ( Figure 2A ). Sanger sequencing verified these results ( Figure 2B ). One variant was a transversion of guanine to cytosine (c.1439+1G>C) in the first base of the 59-donor splice site of intron 9. This variant occurred at a highly conserved “AG-GT” sequence of the exon-intron boundary region. This G>C variant's predicted effect is a disruption of normal splicing, possibly resulting in the retaining of intron 9 of the *SCNN1A* gene. The other heterozygous variant (c. 875+1G> A) has not been reported in the literature and rarely appears in the normal human database gnomAD (1/277126). Software (scSNV) predicts that the intronic variant would affect *SCNN1A* transcript splicing ( Figure 2 ). Based on the expected functions described above and American College of Medical Genetics standards ( 11 ), these two variants were presumptively pathogenic. No other variants were found in the entire exon heterozygous truncation variant and interfered with the protein kinase activity. The variant is not in the Genome Aggregation Database browsers (gnomAD, http://gnomad.broadinstitute.org/ ), the Exome Aggregation Consortium databases (ExAC, http://exac.broadinstitute.org/ ), or dbSNP ( http://www.ncbi.nlm.nih ).

## DISCUSSION

This study describes Chinese fraternal twins who presented to the hospital with similar symptoms, including feeding difficulties, dehydration, and failure to thrive. The twins had salt loss crises within the first to the second week of life. Using clinical trio-exome sequencing, we identified two heterozygous intronic splicing mutants (c.1439+1G>C and c.875+1G>A) in *SCNN1A* gene encoding a sodium channel subunit, which are responsible for PHA1b.

Despite elevated plasma renin and aldosterone levels, systemic PHA1 is mainly characterized by early-onset hyperkalemia, hyponatremia, and severe metabolic acidosis. In these cases, the primary mechanism of hyperreninemia and hyperaldosteronism is a decrease in the volume of extracellular fluid rather than peripheral mineralocorticoid resistance ( 12 – 13 ). Therefore, systemic PHA1 is clinically manifested as feeding difficulties, weight loss, fever, vomiting, diarrhea, recurrent pulmonary infections, dehydration, and life-threatening hyperkalemia, hyponatremia, serve metabolic acidosis, and sudden cardiac arrest ( 14 – 16 ). In our cases, both patients exhibited symptoms of hyponatremia, hyperkalemia, elevated plasma renin activity, and aldosterone levels, which meet the clinical diagnoses of PHA1.

Under normal circumstances, these symptoms in newborns can easily be misdiagnosed as CAH due to deficiency of 21-hydroxylase or 3-beta-hydroxysteroid dehydrogenase and primary hypoaldosteronism causing salt loss during the neonatal period. PHA1 diagnosis is based on elevated plasma aldosterone and renin levels, especially when high-dose mineralocorticoid treatment cannot remedy the imbalance of potassium and salt. Detection of 17-hydroxyprogesterone, ACTH, cortisol, renin, and aldosterone will also provide diagnostic clues to diagnose CAH and PHA1 differentially. Our two patients developed severe shock, electrolyte disturbances, and arrhythmias in the early stages.

The primary treatment for PHA1 is to take high doses of sodium and ion exchange resin and reduce potassium levels through diet control ( 10 ). For some patients, intravenous sodium supplementation and albuterol or glucose/insulin treatment are sufficient to reestablish Na/K balance. If the above treatment fails to achieve normokalaemia, potassium binding resins at high doses may be required. Peritoneal dialysis may be required to correct hyperkalemia ( 17 , 18 ), as in case 2, which required peritoneal dialysis and CRRT to remove serum potassium. Case 2 was treated with Kayexalate to stabilize blood potassium. However, the delay in treatment led to his death by cardiac arrest secondary to hyperkalemia.

**Figure 3:**
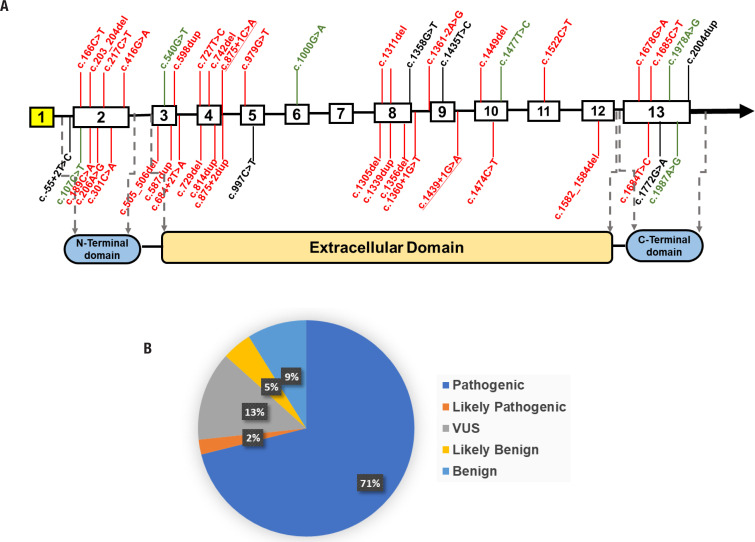
Distribution of variants by location and type. ( **A** ) Schematic diagrams showing the structure of *SCNN1A* A schematic view of the location of PHA1b-related variants in the *SCNN1A* gene. The underlined variants are the variants reported in this study. red: pathogenic and likely pathogenic variants; black: VUS (variants of uncertain significance); green: likely benign and benign variants. ( **B** ) Classification of *SCNN1A* variants associated with PHA1b

Since variant in the *SCNN1* gene was firstly reported to cause PHA1 over two decades ago ( 19 ), nearly 45 SCNN1 variants have been identified, including nonsense/missense variants, splicing variants, small insertions/deletions, and large deletions ( 5 , 16 , 20 ). The *SCNN1A* gene codes the α subunit of the ENaC and is required for channel activity. It plays an essential role in the reabsorption of sodium ions at the collecting ducts of nephrons, the apical membranes of polarized epithelial cells of the colon, sweat glands, and lungs in response to aldosterone ( 16 ). The *SCNN1A* contains 13 exons: exon two codes for the N-terminal domain, exon 13 codes for the C-terminal domain, and exons 3-12 code for the protein's extracellular domain ( Figure 3A ).

We analyzed 45 disease-associated variants obtained from the ClinVar ( https://www.ncbi.nlm.nih.gov/clinvar/ ), LOVD ( https://www.lovd.nl ) database and the Medline search ( Figure 3B , Supplementary Table S1 ). Variants of uncertain significance were found in 6/45 (13%). Likely benign and benign variants were found in 2/45 (5%) and 4/45 (9%), respectively. To date, 71% of the variants have been recognized as pathogenic (32/45), and 2% have been identified as likely pathogenic (1/45) ( Figure 3B ). More than half of the pathogenic and likely pathogenic mutants were found in the 2nd, 3rd, 4th, and 8th exons, indicating structural disruptions with different effects on protein function. Variants in the middle and border regions of the extracellular domain may have a more significant impact on the structure and function of the protein. The UK PHA1 cohort has been reviewed for *SCNN1A* -related PHA1b. The findings suggest that lower serum aldosterone may suggest a missense variant in these patients ( 16 ), consistent with our findings. However, the decrease in aldosterone levels in these patients did not reflect a reduction in clinical severity.

**Table S1:** The list of all reported mutants in *SCNN1A* related to PHA1 systemic type

	Variant	AA change	Classification	Exon/intron
1	c.-55+2T>C	p.?	VUS	1^st^ Intron
2	c.107C>T	p.(Ala36Val)	likely benign	2^nd^ Exon
3	c.166C>T	p.(Arg56*)	pathogenic	2^nd^ Exon
4	c.189C>A	p.(Cys63*)	pathogenic	2^nd^ Exon
5	c.203_204del	p.(Ile68Thrfs*76)	pathogenic	2^nd^ Exon
6	c.206A>G	p.(His69Arg)	pathogenic	2^nd^ Exon
7	c.217C>T	p.(Arg73Cys)	pathogenic	2^nd^ Exon
8	c.301C>A	p.(Gln101Lys)	pathogenic	2^nd^ Exon
9	c.416G>A	p.(Arg139Lys)	pathogenic	2^nd^ Exon
10	c.505_506del	p.(Thr169Serfs*36)	pathogenic	3^rd^ Exon
11	c.540G>T	p.(Leu180=)	benign	3^rd^ Exon
12	c.587dup	p.(Pro197Alafs*9)	pathogenic	3^rd^ Exon
13	c.598dup	p.(Ala200Glyfs*6)	pathogenic	3^rd^ Exon
14	c.684+2T>A	p.?	pathogenic	3^rd^ Intron
15	c.727T>C	p.(Ser243Pro)	pathogenic	4^th^ Exon
16	c.729del	p.(Val245Trpfs*4)	pathogenic	4^th^ Exon
17	c.742del	p.(Val248*)	pathogenic	4^th^ Exon
18	c.814dup	p.(Glu272Glyfs*39)	pathogenic	4^th^ Exon
19	c.875+1C>A	p.?	Likely pathogenic	4^th^ Intron
20	c.875+2dup	p.?	pathogenic	4^th^ Intron
21	c.979G>T	p.(Gly327Cys)	pathogenic	5^th^ Exon
22	c.997C>T	p.(Arg333Cys)	VUS	5^th^ Exon
23	c.1000G>A	p.(Ala334Thr)	benign	6^th^ Exon
24	c.1305del	p.(Tyr436Ilefs*46)	pathogenic	8^th^ Exon
25	c.1311del	p.(Arg438Glyfs*44)	pathogenic	8^th^ Exon
26	c.1339dup	p.(Tyr447Leufs*13)	pathogenic	8^th^ Exon
27	c.1344_1347dup	p.(His450Lysfs*11)	pathogenic	8^th^ Exom
28	c.1356del	p.(Trp453Glyfs*29)	pathogenic	8^th^ Exon
29	c.1358G>T	p.(Trp453Leu)	VUS	8^th^ Exon
30	c.1360+1G>T	p.?	pathogenic	8^th^ Intron
31	c.1361-2A>G	p.?	Pathogenic	8^th^ Intron
32	c.1435T>C	p.(Cys479Arg)	VUS	9^th^ Exon
33	c.1439+1G>C	p.?	Pathogenic	9^th^ Intron
34	c.1449del	p.(Tyr484Thrfs*13)	pathogenic	10^th^ Exon
35	c.1474C>T	p.(Arg492*)	pathogenic	10^th^ Exon
36	c.1477T>C	p.(Trp493Arg)	benign	10^th^ Exon
37	c.1522C>T	p.(Arg508*)	pathogenic	11^th^ Exon
38	c.1582_1584del	p.(Phe528del)	pathogenic	12^th^ Exon
39	c.1678G>A	p.(Gly560Ser)	pathogenic	13^th^ Exon
40	c.1684T>C	p.(Ser562Pro)	pathogenic	13^th^ Exon
41	c.1685C>T	p.(Ser562Leu)	pathogenic	13^th^ Exon
42	c.1772G>A	p.(Arg591Gln)	VUS	13^th^ Exon
43	c.1978A>G	p.(Ser660Gly)	likely benign	13^th^ Exon
44	c.1987A>G	p.(Thr663Ala)	benign	13^th^ Exon
45	c.2004dup	p.(Pro669AlafsTer62)	VUS	13^th^ Exon

In our cases, trio-CES results showed that both patients had compound heterozygous *SCNN1A* variants of c.1439+1G>C and c.875+1G>A. Sanger sequencing verified the paternal origin of c.1439+1G>C and the maternal origin of c.875+1G>A. Both variants identified in our patients are splicing variants in the *SCNN1A* gene. The c.1439+1G>C was reported in one Chinese case with PHA1b before ( 4 ). That case, which was homozygous for c.1439+1 G>C, did not suffer a cardiac arrest. The c.875+1G>A has not been found in published cases. There have been reported five PHA1b cases due to splicing variants in *SCNN1A* (4,7,21-23). The clinical severity differs among these cases and may be attributable to the location of the variants in different exons. Excluding our patients, all the cases with splicing variants in *SCNN1A* showed spontaneous resolution of symptoms, indicating clinical heterogeneity.

In conclusion, we identified two splicing variants in *SCNN1A* in Chinese fraternal twins with PHA1b, including a novel variant. We have provided a review of previous cases to summarize variants in the *SCNN1A* gene, which may be clinically useful. Establishing the diagnosis of PHA1b in our patients emphasizes the value of CES for genetic diagnosis in rare diseases.
